# Prehospital time of suspected stroke patients treated by emergency medical service: a nationwide study in Thailand

**DOI:** 10.1186/s12245-021-00361-w

**Published:** 2021-07-19

**Authors:** Phantakan Tansuwannarat, Pongsakorn Atiksawedparit, Arrug Wibulpolprasert, Natdanai Mankasetkit

**Affiliations:** 1 Chakri Naruebodindra Medical Institute, Faculty of Medicine Ramathibodi Hospital, Mahidol University, Samut Prakan, 10540 Thailand; 2 Department of Emergency Medicine, Faculty of Medicine Ramathibodi Hospital, Mahidol University, Bangkok, Thailand

**Keywords:** Stroke, EMS, Ambulance, Prehospital care, Response time, Thailand

## Abstract

**Background:**

This work was to study the prehospital time among suspected stroke patients who were transported by an emergency medical service (EMS) system using a national database.

**Methods:**

National EMS database of suspected stroke patients who were treated by EMS system across 77 provinces of Thailand between January 1, 2015, and December 31, 2018, was retrospectively analyzed. Demographic data (i.e., regions, shifts, levels of ambulance, and distance to the scene) and prehospital time (i.e., dispatch, activation, response, scene, and transportation time) were extracted. Time parameters were also categorized according to the guidelines.

**Results:**

Total 53,536 subjects were included in the analysis. Most of the subjects were transported during 06.00-18.00 (77.5%) and were 10 km from the ambulance parking (80.2%). Half of the subjects (50.1%) were served by advanced life support (ALS) ambulance. Median total time was 29 min (IQR 21, 39). There was a significant difference of median total time among ALS (30 min), basic (27 min), and first responder (28 min) ambulances, Holm *P* = 0.009. Although 91.7% and 88.3% of the subjects had dispatch time ≤ 1 min and activation time ≤ 2 min, only 48.3% had RT ≤ 8 min. However, 95% of the services were at the scene ≤ 15 min.

**Conclusion:**

Prehospital time from EMS call to hospital was approximately 30 min which was mainly utilized for traveling from the ambulance parking to the scene and transporting patients from the scene to hospitals. Even though only 48% of the services had RT ≤ 8 min, 95% of them had the scene time ≤ 15 min.

**Supplementary Information:**

The online version contains supplementary material available at 10.1186/s12245-021-00361-w.

## Background

Cerebrovascular accident (stroke) is a time-sensitive condition in which blood vessel infarction or hemorrhage causes some disorders to brain function. This is the leading cause of deaths and disabilities worldwide [1]. The prevalence of stroke and stroke-related deaths ranges from 60 to 700 per 100,000 population and from 22.4 to 263.9 per 100,000 population, respectively [1, 2]. To reduce the magnitude of deaths and disabilities, stroke chain of survival has been introduced which includes an early recognition of signs/symptoms of stroke, an activation of emergency medical service (EMS) with timely response, transport to stroke center with pre-arrival notification, and an implementation of guidelines on stroke care with high quality post-stroke rehabilitation [3, 4]. Currently, several pieces of evidence indicate applying EMS system to the stroke care process can improve quality of management and decrease prehospital delays [5–7]. Therefore, the American Heart Association and the American Stroke Association (AHA/ASA) have introduced specific parameters to measure the quality of EMS care for stroke patients which include the highest level of care available for suspected stroke patients: dispatch time ≤ 60 s, activation time ≤ 60 s, response time (RT) ≤ 8 m, and on-scene time ≤ 15 m [8, 9]. In addition, previous studies in several countries, where EMS system is well developed, reported a high percentage (47 to 72%) of stroke patients who were transferred to the hospitals by ambulance [10–12].

In Thailand, the prevalence of stroke is 122 per 100,000 population [13] and this rate in 45 years old or older people contributes 1.88% [14]. This has also been one of the top three causes of burden of disease among Thai population [15]. Stroke fast track protocol has been widely implemented among emergency departments (ED) in Thailand. However, combining EMS system with this protocol has not been systematically initiated because the EMS system is still under the developing stage. Furthermore, previous studies in Thailand reported 5.5 to 20.5% of stroke patients visiting ED by EMS system [16–19]. To identify the strategy for developing EMS stroke fast track, the current performance of EMS on suspected stroke patients should be determined. Hence, we aimed to study the prehospital specific parameters among suspected stroke patients who were transported by EMS system based on the national database.

## Methods

### Study design

We conducted a nationwide cross-sectional study among suspected stroke patients transported to hospitals by EMS system in Thailand between January 1, 2015, and December 31, 2018. This study was approved by the Ethic Committee of the Faculty of Medicine, Ramathibodi Hospital, Mahidol University, Thailand, with a waiving of informed consent.

### Study setting and population

**Fig. 1:**
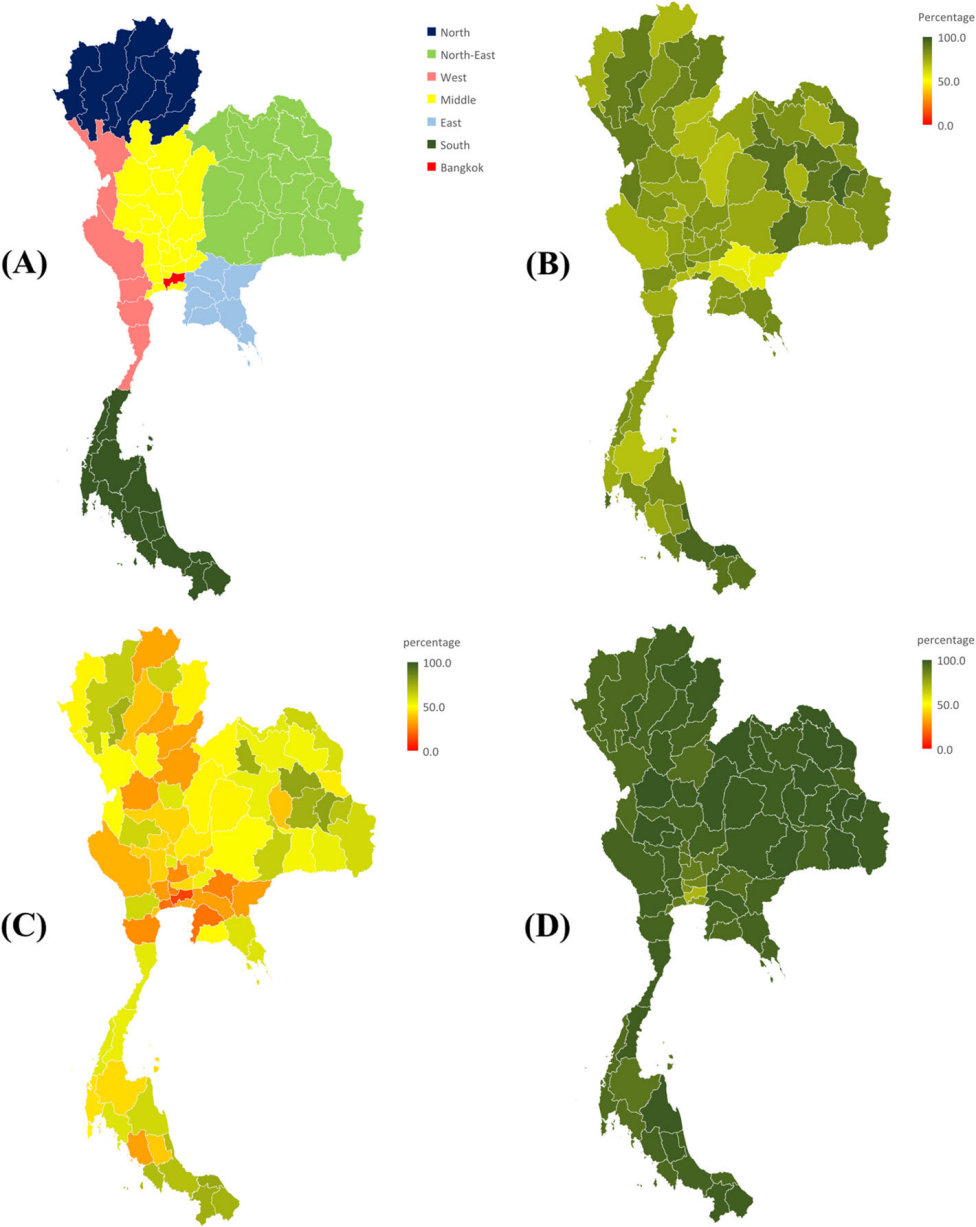
Regions of Thailand and heat map of indicators. (**A**) Regions of Thailand, (**B**) were in 10 km from parking, (**C**) percentage of subjects whose RT ≤ 8 min, and (**D**) percentage of subjects whose scene time ≤ 15 min

In 2019, there were approximately 66.5 million people [20] living in the capital city, Bangkok, and 76 provinces. These 76 provinces are divided into six regions according to the geography (i.e., the north, north-east, middle, east, west, and south), see Fig. 1A [21]. Each province is divided into districts and there are provincial and district-based hospitals.

For a decade, a multi-tiered ground EMS system in Thailand has been established to cover prehospital care. Dispatch centers are located at provincial hospitals. The levels of ambulance are divided into advanced life support (ALS), intermediate life support (ILS), basic life support (BLS), and first responder (FR). ALS ambulances are a fixed deploying model and they are parked at provincial and district hospitals, whereas BLS and FR are fluid deploying models. Dispatch center categorizes chief complaints of all emergency calls regarding 25 criteria-based dispatch. Subsequently, the severity of chief complaints is prioritized to dispatch appropriate levels of ambulance, see Additional file 1 [22]. Emergency level of phone triage is assigned to patients if the gathered information suggests alteration of consciousness, airway obstruction, severe respiratory distress, or shock. For other less severe conditions, urgency level is assigned. In case ALS ambulance is not available, the highest ambulance available is dispatched. Generally, patients are sent to the nearest hospital after prehospital treatment. The information gained during prehospital operation has to be uploaded to the national database via the Information Technology for Emergency Medical System which is managed by the National Institute for Emergency Medicine of the Ministry of Public Health. This study enrolled all suspected stroke adult patients who met criteria-based dispatch code 18: paralysis, weakness, loss of sensation, dysarthria, or facial palsy (ischemic or hemorrhagic stroke), and were transported to hospitals by EMS system from January 1, 2015, to December 31, 2018. We excluded patients whose time variables were missing.

### Variables and data collection

**Fig. 2:**
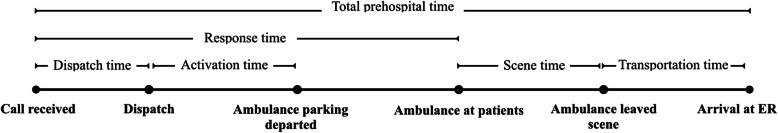
Definition of EMS operation times

All data were retrieved from the Information Technology for Emergency Medical System database. Duplicated records were explored and excluded. Data were cleaned and checked for correctness before the final analysis. The primary outcome was prehospital time intervals (i.e., dispatch, activation, response, scene, and transportation time). The definitions of prehospital time were described in Fig. 2. Dispatch triage was prioritized as emergency and urgency levels.

### Statistical analysis

A complete case analysis was applied. Continuous and categorical data were displayed as median (interquartile range: IQR) and number (%), respectively. According to cut off point of national key performance indicator (KPI), dispatch time, activation time, RT, and distance from EMS base to the scene were categorized to ≤ 1 versus >1 min, ≤ 2 versus >2 min, ≤ 8 versus > 8 min, and ≤ 10 versus > 10 km, respectively [23]. In contrast, the scene time was categorized as ≤ 15 versus >15 min based on AHA/ASA 2007 and 2013 recommendations [8, 9]. The Chi-square test was used to compare the difference among categorical data. For continuous data, the test for normality was performed prior to subsequent analysis. Linear regression or quantile regression was used to compare more than two groups of normal distributed and non-normal distributed continuous data, respectively. An adjustment for multiple hypothesis testing was applied by estimating the family-wise error rate (Holm *P* value) by using the Holm-Bonferroni correction [24]. Statistical significance was considered if Holm *P* value < 0.05. All analyses were conducted using STATA version 15.0 (Stata Corp., College Station, TX, USA), except for map charts which were constructed by Microsoft Excel (2019).

## Results

During the study period, there were 55,372 suspected stroke subjects transported to the emergency room by ambulance. Of those, 1836 (3.3%) subjects were excluded due to incomplete information. Therefore, 53,536 subjects were included in the final analysis.

### Characteristics of EMS operation

Most of the subjects were in the north-eastern region (38.9%). Approximately, 77.5% of the subjects were transported during 06.00-18.00. Only half of the subjects were served by ALS ambulances (50.1%) and prioritized as emergency level (52.2%). There were 80.2% of the subjects who were within 10 km from the ambulance parking. The characteristics of subjects from 2015 to 2018 were consistent, see Table 1.

**Table 1:** Characteristics of suspected stroke patients who were transported to hospital by ambulance between 2015 and 2018

Characteristics	Total		Years							
			2015		2016		2017		2018	
	*n*	(%)	*n*	(%)	*n*	(%)	*n*	(%)	*n*	(%)
	53,536	(100)	9829	(100)	11,844	(100)	14,456	(100)	17,407	(100)
Regions										
North	4736	(8.8)	764	(7.8)	1088	(9.2)	1306	(9)	1578	(9.1)
North-East	20,831	(38.9)	4100	(41.7)	4565	(38.5)	5645	(39)	6521	(37.5)
West	3587	(6.7)	684	(7)	815	(6.9)	895	(6.2)	1193	(6.9)
Middle	9926	(18.5)	1761	(17.9)	2085	(17.6)	2761	(19.1)	3319	(19.1)
East	3482	(6.5)	583	(5.9)	776	(6.6)	949	(6.6)	1174	(6.7)
South	8072	(15.1)	1523	(15.5)	1752	(14.8)	2140	(14.8)	2657	(15.3)
Bangkok	2902	(5.4)	414	(4.2)	763	(6.4)	760	(5.3)	965	(5.5)
Shift										
06.00-18.00	41,476	(77.5)	7634	(77.7)	9169	(77.4)	11,264	(77.9)	13,409	(77)
18.00-06.00	12,060	(22.5)	2195	(22.3)	2675	(22.6)	3192	(22.1)	3998	(23)
Levels										
ALS	26,813	(50.1)	4855	(49.4)	5910	(49.9)	7336	(50.7)	8712	(50)
ILS and BLS	8502	(15.9)	1718	(17.5)	1877	(15.8)	2135	(14.8)	2772	(15.9)
FR	18,221	(34)	3256	(33.1)	4057	(34.3)	4985	(34.5)	5923	(34)
Dispatch triage										
Emergency	27,940	(52.2)	5041	(51.3)	6157	(52)	7641	(52.9)	9101	(52.3)
Urgency	25,596	(47.8)	4788	(48.7)	5687	(48)	6815	(47.1)	8306	(47.7)
Distance (km), median (IQR)	5	(2, 9)	5	(2, 9)	5	(2, 9)	5	(2, 9)	5	(2, 9)
≤ 10 km	42,921	(80.2)	7893	(80.3)	9563	(80.7)	11,541	(79.8)	13,924	(80)
> 10 km	10,615	(19.8)	1936	(19.7)	2281	(19.3)	2915	(20.2)	3483	(20)
Dispatch time										
> 1 min	4417	(8.3)	726	(7.4)	1018	(8.6)	1131	(7.8)	1542	(8.9)
≤ 1 min	49,119	(91.7)	9103	(92.6)	10,826	(91.4)	13,325	(92.2)	15865	(91.1)
Activation time										
> 2 min	6262	(11.7)	1038	(10.6)	1368	(11.6)	1698	(11.7)	2158	(12.4)
≤ 2 min	47,274	(88.3)	8791	(89.4)	10,476	(88.4)	12,758	(88.3)	15,249	(87.6)
Response time										
> 8 min	27,661	(51.7)	4900	(49.9)	6009	(50.7)	7578	(52.4)	9174	(52.7)
≤ 8 min	25,875	(48.3)	4929	(50.1)	5835	(49.3)	6878	(47.6)	8233	(47.3)
Scene time										
> 15 min	2682	(5.0)	419	(4.3)	646	(5.5)	732	(5.1)	885	(5.1)
≤ 15 min	50,854	(95.0)	9410	(95.7)	11,198	(94.5)	13,724	(94.9)	16,522	(94.9)

*ALS* advanced life support, *BLS* basic life support, *FR* first responder, *ILS* intermediate life support, *IQR* interquartile range

Table 2 described the number (%) of the subjects who were ≤ 10 versus > 10 km from the ambulance parking. The results indicated the percentage ranged from 70 to 84.7% across six regions, whereas only 66.3% was found in Bangkok. There was a significantly different percentage of the subjects who were 10 km away from the ambulance parking with FR, BLS, and ALS ambulances contributing 90.4%, 83%, and 72.3%, respectively (Holm *P* = 0.039).

**Table 2:** Number of subjects who were far from parking ≤ 10 km versus >10 km

Factors	*N*	≤ 10 km		> 10 km		*P* value	Holm *P* value
		*n*	(%)	*n*	(%)		
Regions							
North	4736	3827.00	(80.8)	909	(19.2)	< 0.001	0.020
North-East	20,831	17,291.00	(83)	3540.00	(17)		
West	3587	2820.00	(78.6)	767	(21.4)		
Middle	9926	7778.00	(78.4)	2148.00	(21.6)		
East	3482	2439.00	(70)	1043.00	(30)		
South	8072	6841.00	(84.7)	1231.00	(15.3)		
Bangkok	2902	1925.00	(66.3)	977	(33.7)		
Shifts							
06.00-18.00	41,476	32,908.00	(79.3)	8568.00	(20.7)	< 0.001	0.009
18.00-06.00	12,060	10,013.00	(83)	2047.00	(17)		
Levels							
ALS	26,813	19,395.00	(72.3)	7418.00	(27.7)	< 0.001	0.039
ILS and BLS	8502	7053.00	(83)	1449.00	(17)		
FR	18,221	16,473.00	(90.4)	1748.00	(9.6)		
Dispatch triage							
Emergency	27,940	20,846.00	(74.6)	7094.00	(25.4)	< 0.001	0.029
Urgency	25,596	22,075.00	(86.2)	3521.00	(13.8)		

*ALS* advanced life support, *BLS* basic life support;,*FR* first responder, *ILS* intermediate life support

The characteristics of EMS operation (i.e., phone triage and levels of ambulance) among six regions and Bangkok were different (Holm *P* = 0.009 and 0.019, respectively), see Table 3. The percentage of the subjects who were prioritized as emergency level ranged from 33.3 to 85.1%. The higher percentage of emergency cases and the higher percentage of ALS ambulances were deployed. Additionally, there was no FR ambulance dispatched to subjects in Bangkok.

**Table 3:** Characteristics of EMS operation based on regions

Characteristics	Regions							*P* value	Holm *P* value
	North	North-East	West	Middle	East	South	Bangkok		
	*n* (%)	*n* (%)	*n* (%)	*n* (%)	*n* (%)	*n* (%)	*n* (%)		
Phone triage									
Emergency	2126 (55.6)	5759 (33.3)	1347 (47.8)	5266 (67.7)	1436 (58.9)	3273 (47.8)	1639 (85.1)	< 0.001	0.009
Urgency	1701 (44.4)	11,532 (66.7)	1473 (52.2)	2512 (32.3)	1003 (41.1)	3568 (52.2)	286 (14.9)		
Levels									
ALS	1883 (49.2)	5204 (30.1)	1520 (53.9)	5085 (65.4)	1411 (57.9)	2674 (39.1)	1618 (84.1)	< 0.001	0.019
ILS and BLS	642 (16.8)	2733 (15.8)	657 (23.3)	1038 (13.3)	94 (3.9)	1582 (23.1)	307 (15.9)		
FR	1302 (34)	9354 (54.1)	643 (22.8)	1655 (21.3)	934 (38.3)	2585 (37.8)	0		

*ALS* advanced life support, *BLS* basic life support, *FR* first responder, *ILS* intermediate life support

### EMS operation times

EMS operation time was described in Table 4. Median total prehospital time among enrolled subjects was 29 min (IQR 21, 39). The longest total time was found in an advanced level with median 30 min (Holm *P* = 0.014). Most of the total time was occupied by transportation time (median 10 min with IQR 6, 17), response time (median 9 min with IQR 5, 14), and scene time (median 5 min with IQR 2, 8), respectively. Median response time of FR was significantly shorter than that of ILS/BLS and ALS ambulances (6 min versus 8 and 11 min, respectively; Holm *P* = 0.029). However, median scene time of FR (3 min) was also shorter than that of ILS/BLS and ALS ambulances (4 and 5 min, respectively; Holm *P* = 0.024), but median transportation time was longer (14 min versus 10 and 9 min, respectively; Holm *P* = 0.019).

**Table 4:** EMS operations times among suspected stroke patients based on levels of ambulance

Times	Overall	Levels of ambulance, median (IQR)				Holm *P* value
	median (IQR)	FR	ILS/BLS	Advance	*P* value	
Dispatch time	1 (1, 1)	1 (1, 1)	1 (1, 1)	1 (1, 1)	< 0.001	0.005
Activation time	1 (1, 2)	1 (1, 1)	1 (0, 1)	1 (1, 2)	< 0.001	0.010
Response time	9 (5, 14)	6 (4, 10)	8 (5, 13)	11 (7, 17)	< 0.001	0.029
Scene time	5 (2, 8)	3 (2, 5)	4 (2, 7)	5 (3, 10)	< 0.001	0.024
Transportation time	10 (6, 17)	14 (8, 21)	10 (5, 15)	9 (5, 14)	< 0.001	0.019
Total prehospital time	29 (21, 39)	28 (20, 37)	27 (19, 36)	30 (21, 42)	< 0.001	0.014
Times	Overall	Levels of ambulance, *n* (%)				Holm *P* value
	*n* (%)	FR	ILS/BLS	Advance	*P* value	
Dispatch time						
>1 min	4417 (8.3)	768 (4.2)	636 (7.5)	3013 (11.2)	< 0.001	0.029
≤1 min	49,119 (91.7)	17,453 (95.8)	7866 (92.5)	23,800 (88.8)		
Activation time						
>2 min	6262 (11.7)	1240 (6.8)	595 (7)	4427 (16.5)	< 0.001	0.039
≤2 min	47,274 (88.3)	16,981 (93.2)	7907 (93)	22,386 (83.5)		
Response time						
>8 min	27,661 (51.7)	5967 (32.7)	3975 (46.8)	17,719 (66.1)	< 0.001	0.049
≤8 min	25,875 (48.3)	12,254 (67.3)	4527 (53.2)	9094 (33.9)		
Scene time						
>15 min	2682 (5)	378 (2.1)	291 (3.4)	2013 (7.5)	< 0.001	0.019
≤15 min	50,854 (95)	17,843 (97.9)	8211 (96.6)	24,800 (92.5)		

*ALS* advanced life support, *BLS* basic life support, *FR* first responder, *ILS* intermediate life support

There were 91.7% and 88.3% of the operations had dispatch time in 1 min and activation time in 2 min, respectively. Despite this, only 48.3% of the operations had RT ≤ 8 min. However, most of the operations (95%) were at the scene in less than 15 min. The lowest percentage of dispatch time ≤ 1 min (88.8%), activation time ≤ 2 min (83.5%), response time ≤ 8 min (33.9%), and scene time ≤ 15 min (92.5%) were observed in an advanced ambulance (Holm *P* = 0.029, 0.039, 0.049, and 0.019, respectively).

The results indicated the percentage of RT ≤ 8 min ranged from 13.1 to 56.6% across six regions. The highest percentage was found in the north-east, whereas the lowest percentage was found in Bangkok. Operations during 18.00-06.00 achieved RT ≤ 8 min more than during 06.00-18.00 (51.2% versus 47.5%, Holm *P* = 0.009). Operations by FR had the highest percentage of RT ≤ 8 min, compared to BLS and ALS teams with 67.3% versus 53.3% and 33.9%, respectively (Holm *P* = 0.039). Operations which were prioritized as emergency level had a lower percentage of RT ≤ 8 min, compared to urgent cases (38.2% versus 59.4%, Holm *P* = 0.029). There was a higher percentage of RT ≤ 8 min when considering only subjects who were within 10 km from the ambulance parking, see Table 5.

**Table 5:** Number of RT ≤ 8 min among overall and subjects in 10 km from parking

Factors	Overall (*N* = 53,536)					*P* value	Holm P value	≤ 10 km distance (*N* = 42,921)					*P* value	Holm *P* value
	Total	≤ 8 min		> 8 min				Total	≤ 8 min		> 8 min			
		*n*	%	*n*	%				*n*	%	*n*	%		
Region														
North	4736	2172	(45.9)	2564	(54.1)	< 0.001	0.019	3827	2134	(55.8)	1693	(44.2)	< 0.001	0.039
North-East	20,831	11,799	(56.6)	9032	(43.4)			17,291	11,606	(67.1)	5685	(32.9)		
West	3587	1654	(46.1)	1933	(53.9)			2820	1624	(57.6)	1196	(42.4)		
Middle	9926	4000	(40.3)	5926	(59.7)			7778	3927	(50.5)	3851	(49.5)		
East	3482	1270	(36.5)	2212	(63.5)			2439	1234	(50.6)	1205	(49.4)		
South	8072	4601	(57)	3471	(43)			6841	4519	(66.1)	2322	(33.9)		
Bangkok	2902	379	(13.1)	2523	(86.9)			1925	331	(17.2)	1594	(82.8)		
Shifts														
06.00-18.00	41,476	19,696	(47.5)	21,780	(52.5)	< 0.001	0.009	32,908	19,300	(58.6)	13,608	(41.4)	< 0.001	0.029
18.00-06.00	12,060	6179	(51.2)	5881	(48.8)			10,013	6075	(60.7)	3938	(39.3)		
Levels														
ALS	26,813	9094	(33.9)	17,719	(66.1)	< 0.001	0.039	19,395	8859	(45.7)	10,536	(54.3)	< 0.001	0.019
BLS	8502	4527	(53.2)	3975	(46.8)			7053	4446	(63)	2607	(37)		
FR	18,221	12,254	(67.3)	5967	(32.7)			16,473	12,070	(73.3)	4403	(26.7)		
Dispatch triage														
Emergency	27,940	10,667	(38.2)	17,273	(61.8)	< 0.001	0.029	20,846	10,399	(49.9)	10,447	(50.1)	< 0.001	0.009
Urgency	25,596	15,208	(59.4)	10,388	(40.6)			22,075	14,976	(67.8)	7099	(32.2)		

*ALS* advanced life support, *BLS* basic life support, *FR* first responder, *ILS* intermediate life support

Figure 1 showed a heat map of the percentage of the subjects who were within 10 km from the ambulance parking (B), RT ≤ 8 min (C), and scene time ≤ 15 min (D), across 76 provinces and Bangkok. The percentage of the subjects who were within 10 km in most provinces of the northern, north-eastern, and southern regions were higher than those in the middle, eastern, western regions, and Bangkok, see Fig. 1B. Moreover, most provinces had a low percentage of RT ≤ 8 min, especially those in the west, the lower part of the middle, the east, and Bangkok, see Fig. 1C. However, all provinces had a high percentage of the scene time ≤ 15 min, except for Bangkok and the vicinity, see Fig. 1D.

## Discussion

Analysis of the national database showed that only half of the suspected stroke patients in Thailand were transported by ALS ambulances. A median total prehospital time was approximately 30 min which was mainly spent on transportation, response, and scene time. Nevertheless, there was a good performance of dispatch, activation, and scene time, but only half of the operations met the target KPI of RT.

The current recommendation for prehospital management for suspected stroke patients includes an early recognition of signs/symptoms, an immediate activation of EMS system, a response with high level EMS ambulance, an application of prehospital stroke screening tools and rapid transport of the patients to stroke centers [3, 25–28]. Several important findings were found:

First, only half of the suspected stroke subjects who called the EMS system were prioritized as emergency level (52.5%), and transported to the receiving hospitals by ALS ambulances (50.1%). The percentage was much lower than that in the previous studies in the developed countries which were 60 to 89% [29–31]. These findings could be explained by the fact that the stroke fast track protocol is not implemented on EMS system. Suspected stroke patients are not immediately prioritized to be high level of triage but they are mainly prioritized based on life-saving conditions, e.g., alteration of consciousness or breathing problems. In addition, there might be limited numbers and distributions of ALS ambulances across Thailand. As a result, lower level ambulances are deployed instead.

Second, we found a huge gap between the proportion of emergency level of phone triage and the proportion of ALS ambulances serving suspected stroke patients among different regions despite a widespread use of emergency medical triage and criteria-based protocols. This problem suggests that the mentioned protocols might not be effectively implemented.

Third, there were a higher number of patients transported to hospitals in day time, compared to night time. This might be explained by the symptoms of stroke that could be detected while the patients have full consciousness. In general, most of the patients observe the abnormalities after waking up or during the daytime and subsequently call for help.

According to the mentioned findings, assigning suspected stroke patients as a high level of triage is important and should be emphasized in stroke fast tract protocol. Besides, regular triage audits should be conducted aiming to explore and sustain the standardization of triage protocol used among EMS personnel. In addition, a number of patients could be used to determine an efficient allocation of staff. However, training EMT and FR to assess stroke signs/symptoms under the supervision of standardized direct medical command via tele-consultation might be an area for improvement if increasing the number of ALS ambulances is difficult.

The results showed median total prehospital time was approximately 30 min which correlated with the previous studies [29–35]. Our results also revealed a high percentage of dispatch ≤ 1 min and activation ≤ 2 min [23, 25]. However, our median RT was longer than that in the recommendation and other studies [3, 25, 27, 29–32, 34], and only half of the subjects experienced RT ≤ 8 min. Nonetheless, short dispatch and activation time pointed out that prompt ambulances were available, but long RT also indicated that ambulances took a long time to reach patients. This might be the result of long distance from the ambulance parking to the scene (Tables 1 and 2), traffic, and geographical problems, e.g., mountainous or rural areas.

Advanced ambulances had the lowest percentage of RT ≤ 8 min, compared to lower level ambulances. This might be explained by different deployment models between ALS versus FR/BLS ambulances in Thailand. ALS ambulances are parked at hospitals as a fixed deployment model, while FR/BLS ambulances are available in the most frequent scene areas as a fluid deployment model. For this reason, the FR/BLS could reach the patients faster but spend longer time transporting patients to hospitals. Therefore, exploring patient distribution in each geographical area and strengthening the collaboration between FR/BLS and advanced ambulances might be required to improve the reallocation of EMS service for stroke patients. Furthermore, redesigning a coordinated system between FR/BLS and ALS ambulances to provide stroke care may be suitable for Thailand’s context.

Most of the total prehospital time was spent on traveling from parking to the scene and from the scene to hospital, which differed from that in the previous studies in developed countries where most of the prehospital time is spent at the scene [30, 31, 34]. EMS system in Thailand is a scoop and run model in which patients are initially evaluated and provided with necessary medical treatments at the scene, before being transported to the nearest hospital. Most interventions for stroke protocol (e.g., EKG, intravenous assessment, and blood collection) are primarily performed at ED. Therefore, our scene time was very short. From this, implementing stroke screening by EMS personnel and activating stroke fast track team before the patients arrive at ED might be the options to improve stroke chain of survival [3, 25–27].

In Bangkok, 85% of the cases was transported by ALS ambulances which was very high, compared to other regions. This might be because FR ambulances are not available. Thus, ALS ambulances are activated in high proportion. There are a large number of both public and private hospitals in Bangkok, compared to the upcountry. Nevertheless, the uneven hospital distribution and the complexity of the capital city (e.g., dense population, houses, or traffic jams) could explain why only 13% of the operations had RT ≤ 8 min.

The strength of this study was that we used the national database which represents all EMS operations across Thailand. In addition, this data set contains a low amount of missing time information and this decreased selection bias. However, some limitations were also identified. First, this database includes only prehospital information. As a result, we were unable to assess other clinical important factors (e.g., facilities of the receiving hospitals and patients’ final diagnosis and outcomes). For this reason, the development of prehospital stroke care should include a comprehensive database linked from prehospital to hospital phases. Second, we were unable to estimate the proportion of patients self-transporting to the hospital because we included only the subjects who were transported by the ambulance. Although this proportion was not estimated, previous studies reported less than 20% of Thai stroke patients visiting ED by EMS system [16–19]. Therefore, an emphasis on people’s education about stroke recognition and an access to EMS service should be included into the protocol.

Based on our results, several strategies should be considered for implementing prehospital stroke care in Thailand as follows:

1. People should be educated about early stroke recognition (e.g., signs or symptoms of stroke) and an access to EMS service. These will boost an early recognition and immediate activation of EMS system.
2. Phone triage protocol should be revised by increasing the level of triage among suspected stroke patients and responding with a high level of ambulance.
3. EMS personnel should be empowered and trained with prehospital stroke screening tools which aim to transport the right patient to the right receiving hospital.
4. The coordination system between FR, BLS, and ALS ambulances in taking care of stroke patients and taking direct medical command via tele-consultation might be appropriate for Thailand, where an increase in ALS units is difficult.
5. A comprehensive and reliable stroke registry system linking from prehospital to hospital phase should be developed. This will help the system to determine several dimensions of stroke care, e.g., time interval, appropriateness of prehospital/in hospital treatment, appropriateness of the receiving hospitals, a final diagnosis, and patient outcomes. Finally, regular system audits should be conducted to monitor and improve stroke care.

## Conclusions

In summary, this study demonstrated that prehospital time from the receiving EMS call to the patient’s arrival at ER was approximately 30 min. This time interval was mainly spent on traveling from the ambulance parking to the scene and transporting patients from the scene to ER. Only 48% of the total operations had RT ≤ 8 min, but most of them (95%) had the scene time ≤ 15 min.

## Supplementary Information

**Additional file 1:.** 25 chief complaints of emergency medical triage protocol and criteria-based dispatch 2013.

## Data Availability

The datasets and/or analyzed during the current study are available from the corresponding author on reasonable request.

## Abbreviations

ALS: Advanced life support
BLS: Basic life support
ED: Emergency department
EMS: Emergency medical service
EMT: Emergency medical technician
FR: First responder
ILS: Intermediate life support
IQR: Interquartile range
KPI: Key performance indicator
RT: Response time
