# Supplementary material for: Prehospital time of suspected stroke patients treated by emergency medical service: a nationwide study in Thailand
Source: Int J Emerg Med. 2021 Jul 19;14:37. doi: 10.1186/s12245-021-00361-w (PMC8287686; doi:10.1186/s12245-021-00361-w)
Supplement: Supplementary file 1 — Additional file 1:. 25 chief complaints of emergency medical triage protocol and criteria-based dispatch 2013. [file 12245_2021_361_MOESM1_ESM.docx]

Additional file 1: 25 chief complaints of emergency medical triage protocol and criteria-based dispatch 2013

| Types | codes | Chief complaints |
| --- | --- | --- |
| Non-trauma | 1 | Pain including abdomen, back, pelvic area |
|  | 2 | Anaphylaxis/ Allergy reaction |
|  | 3 | Animal bite |
|  | 4 | Non-traumatic hemorrhage |
|  | 5 | Dyspnea |
|  | 6 | Cardiac arrest |
|  | 7 | Chest pain/ discomfort |
|  | 8 | Airway obstruction |
|  | 9 | Diabetes |
|  | 10 | Environmental injuries |
|  | 11 | - |
|  | 12 | Pain including head and neck |
|  | 13 | Psychosis/ emotional problems |
|  | 14 | Drug overdose/ intoxication |
|  | 15 | Pregnancy/ Gynecologic problems |
|  | 16 | Seizures |
|  | 17 | Fever/ fatigue |
|  | 18 | paralysis/weakness/loss of sensation/dysarthria /facial palsy (ischemic or hemorrhagic stroke) |
|  | 19 | Syncope |
|  | 20 | Pediatric problems |
| Trauma | 21 | Trauma/ Assaults |
|  | 22 | Burn |
|  | 23 | Drowning/Diving injury/Marine injury |
|  | 24 | Falling |
|  | 25 | Motor-vehicle injury |
